# Anti-diabetic activity of stigmasterol from soybean oil by targeting the GLUT4 glucose transporter

**DOI:** 10.1080/16546628.2017.1364117

**Published:** 2017-08-23

**Authors:** Jialin Wang, Mi Huang, Jie Yang, Xinhua Ma, Sijian Zheng, Shihao Deng, Yun Huang, Xinzhou Yang, Ping Zhao

**Affiliations:** ^a^ School of Pharmaceutical Sciences, South-Central University for Nationalities, Wuhan, China; ^b^ National Demonstration Center for Experimental Ethnopharmacology Education, South-Central University for Nationalities, Wuhan, China; ^c^ College of Life Sciences, South-Central University for Nationalities, Wuhan, China

**Keywords:** Anti-diabetic agents, stigmasterol, L6 cells, KK-Ay mice, insulin resistance, glucose transporter 4 (GLUT4)

## Abstract

The present study investigated the anti-diabetic activity and potential mechanism of stigmasterol (SMR), which is a kind of phytosterols derived from the edible soybean oil *in vitro* and *in vivo*. SMR displayed a mild GLUT4 translocation activity by 1.44-fold in L6 cells. L6 cells were treated with different concentration of SMR, showing significant effects on the enhancing glucose uptake. SMR administrated orally to the KK-Ay mice significantly alleviated their insulin resistance and oral glucose tolerance with reducing fasting blood-glucose levels and blood lipid indexes such as triglyceride and cholesterol. Moreover, the GLUT4 expression in L6 cells, skeletal muscle and white adipose tissue had been also enhanced. In this paper we conclude that, stigmasterol seems to have potential beneficial effects on the treatment of type 2 diabetes mellitus with the probable mechanism of targeting GLUT4 glucose transporter included increasing GLUT4 translocation and expression.

## Graphical Abstract

**Figure:**
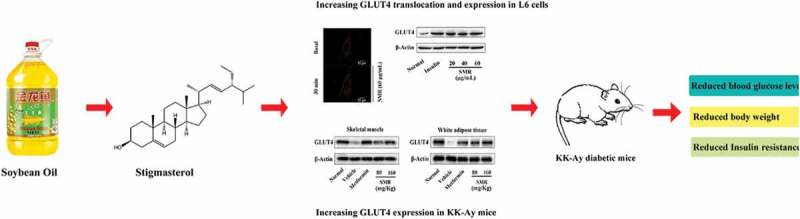

## Introduction

Diabetes mellitus (DM) is one of most common chronic metabolic diseases in almost all countries across the world. It occurs either when the pancreas does not produce enough insulin or when the body cannot effectively utilize the insulin that is produced [1]. Excessive blood glucose, which is often seen as the main characteristic of DM, is sometimes accompanied by lipids and protein metabolism disorder [2]. With the rapid development of society and changes in people’s lifestyle, DM has become the third biggest threat to human health after cardiovascular and neoplastic diseases, according to the World Health Organization (WHO) [3]. There are two types of diabetes: type 1 diabetes (insulin-dependent) and type 2 diabetes (non-insulin-dependent). The most common form is type 2 diabetes mellitus (T2DM), which up to 90% of diagnosed DM patients have [4].

T2DM is a progressive disease and its typical clinical manifestations include a gradual decline in glycemic control caused by insulin resistance (IR) and β cell functional deterioration [5]. The primary cause of T2DM is obesity-driven IR in the liver, white adipose tissue (WAT), and skeletal muscle, combined with the relative insufficient secretion of insulin by pancreatic β cells [6]. At present, the available drugs for T2DM fall into four general categories: insulin, insulin secretagogues, insulin sensitizers, and prandial glucose regulators [7]. The drugs currently available have only limited efficacy, limited tolerability, and significant mechanism-based side effects. Therefore, looking for safer, more effective drugs for T2DM is becoming an urgent problem.

Natural plant remedies are a new trend in modern clinical medicine, but have a long history of use as alternative treatments for T2DM, in which they have shown the satisfactory efficacy and few side effects. Extracts and natural products derived from edible plants, such as *Momordica charantia, Punica granatum, Psidium guajava*, and *Lycium chinensis* have drawn more and more attention because of their safety profile and for the significant anti-diabetic effects that have been observed in previous studies [8–11]. Natural plant remedies from readily available edible plants have great potential for the prevention and treatment of T2DM.

**Figure 1.**
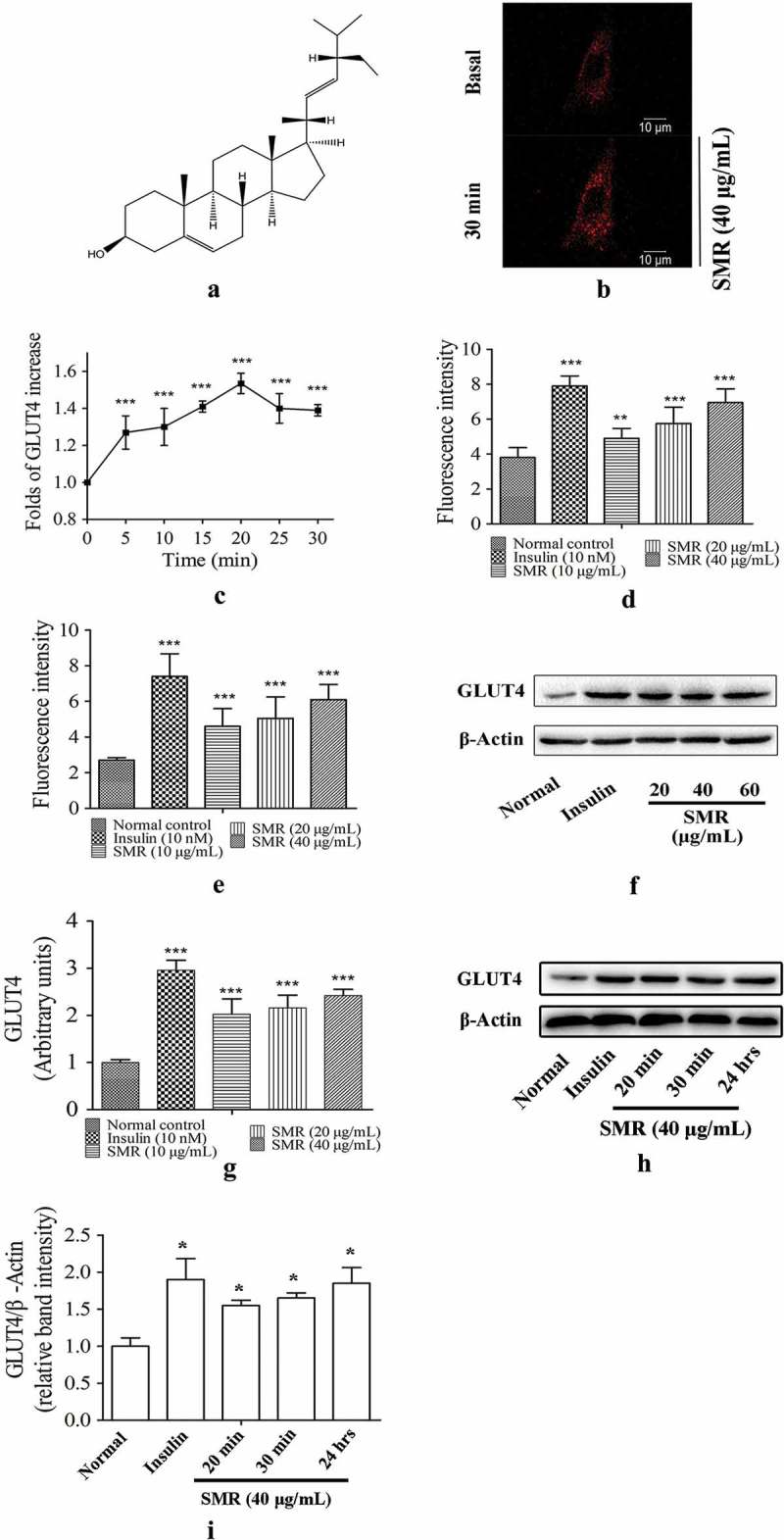
Structure of SMR and the effects of SMR on GLUT4 expression and translocation in L6 cells. (a) The chemical structure of SMR. (b) Confocal images of SMR stimulating the GLUT4 translocation in L6 cells. (c) Data represent the fold increase in fluorescence induced by SMR between 0 and 30 min (***p < 0.001 compared with basal group). (d) Effects of SMR on increasing glucose uptake in L6 cells (**p < 0.01, ***p < 0.001 compared with normal control). (e) Effects of SMR on increasing glucose uptake in 3T3-L1 cells (***p < 0.001 compared with normal control). (f) Effects of SMR on GLUT4 expression in L6 cells. (g) Relative band intensity of GLUT4 in each group (***p < 0.001 compared with normal control).

Phytosterol is a bioactive natural product present in plants that has a structure similar to cholesterol, has no influence on human cholesterol (TC) and low-density lipoprotein cholesterol (LDL-C), and increases the ratio of HDL-C/LDL-C and HDL-C/TC in blood [12]. As a member of the phytosterol family, stigmasterol (SMR) (Figure 1(a)) is widely present in plant oil and plant-based foods, such as soybean, corn, peanut, and sunflower oils [13]. Previous studies have reported that SMR has a variety of pharmacological properties, including anti-osteoarthritic, anti-mutagenic, anti-inflammatory activity, and anti-tumor properties [14–17]. One recent work, reported that a banana extract containing 21.91% SMR exhibited a potential anti-diabetic effect in alloxan-induced diabetic rats [18]; however, the anti-diabetic effects and underlying molecular mechanism of SMR still remain unclear.

In the present study, we explored the anti-diabetic effects of SMR by conducting an experimental investigation in L6 cells and in KK-Ay mice. We also elucidated its probable anti-diabetic molecular mechanism. In an *in vitro* study, SMR exhibited mild glucose transporter 4 (GLUT4) translocation activity when screened with a cell-based GLUT4 translocation assay in L6 cells that stably express pIRAP-mOrange. Corresponding to the increased GLUT4 translocation in the L6 cells, SMR also visibly promoted glucose uptake in the L6 cells. In an *in vivo* study, SMR displayed significant effects on defecting IR and improving oral glucose tolerance in KK-Ay mice. At the same time, the reduced fasting blood glucose levels and blood lipid indexes such as triglyceride and cholesterol were also observed in SMR-treated KK-Ay mice. A western blot assay showed that SMR improved GLUT4 expression in L6 cells, skeletal muscle, and WAT. In general, it seems that SMR displays potential as a treatment for T2DM and the mechanism involves targeting a GLUT4 glucose transporter.

## Materials and methods

### Instruments and reagents

The NMR spectra were recorded on the Bruker DRX-600 NMR spectrometer for ^1^H- and ^13^C-NMR. EI-MS data were obtained on a Finnigan-MAT-95 mass spectrometer. Preparative isolations were performed on Agela HP P050 medium pressure column chromatography (MPCC) system (Tianjin, China). Commercial silica gel (Qing Dao Hai Yang Chemical Group Co., 300–400 mesh) was used for column chromatography. Pre-coated silica gel plates (Yan Tai Zi Fu Chemical Group Co., G60 F-254) were used for analytical thin layer chromatography (TLC). All the solvents used for column chromatography were of analytical-reagent grade.

### Material and SMR preparation

The commercially available soybean oil (Jing Long Yu) was purchased from Wal-Mart supermarket in September 2015 in Wuhan, Hubei province, China. The soybean oil (300 g) was subject to MPCC on silica gel (300–400 mesh, 1500 g), using a gradient of cyclohexane–ethyl acetate (30:1 → 1:2) as eluent, to yield seven fractions. Fraction 4 (43.5 g) was subjected to repeat MPCC on silica gel (300–400 mesh, 800 g) eluted by a gradient of cyclohexane–acetone (20:1 → 7:1) to give seven fractions Fr 4.1–Fr 4.7. Seven fractions were detected compared with the SMR standard by the silica gel TLC (developing solvent, cyclohexane–acetone 10:1), indicating that Fr 4.4 mainly contained SMR. Fr 4.4 (7.9 g) was dissolved in anhydrous acetone (150 mL) to yield 4.5 g of SMR by repeated crystallization at 4°C. SMR was analyzed for purity by analytical C_18_ HPLC (200 nm) and shown to be > 98%.

### Chemical elucidation of SMR

The isolated compound was identified as SMR by comparison with the mass spectrum and NMR spectra with the published reference data [19]. SMR: white amorphous powder; ^1^H NMR (CDCl_3_, 600 MHz): δ_H_ 0.67 (3H, s, H-18), 0.78 (3H, s, H-27), 0.79 (3H, s, H-19), 0.81 (3H, s, H-29), 0.82 (3H, s, H-26), 1.02 (3H, brs, H-21), 3.36 (1H, m, H-3α), 5.12 (1H, dd, *J* = 15.4, 8.4, H-22), 5.33 (1H, d, *J *= 4.7 Hz, H-6); ^13^C NMR (CDCl_3_, 150 MHz): δ_C_ 37.3 (C-1), 31.8 (C-2), 71.8 (C-3), 41.3 (C-4), 140.7 (C-5), 121.7 (C-6), 31.8 (C-7), 31.9 (C-8), 50.2 (C-9), 36.6 (C-10), 21.2 (C-11), 39.8 (C-12), 42.3 (C-13), 56.9 (C-14), 24.3 (C-15), 29.1 (C-16), 56.1 (C-17), 11.9 (C-18), 19.4 (C-19), 40.5 (C-20), 21.1 (C-21), 138.3 (C-22), 129.2 (C-23), 51.2 (C-24), 31.7 (C-25), 19.0 (C-26), 21.2 (C-27), 25.4 (C-28), 12.0 (C-29); EIMS: 412 M^+^. NMR and mass spectra of SMR could be found in S2–S4 in Supplementary Material.

### Cell culture

Rat L6 cells were maintained in a humidiﬁed incubator at 37°C with ambient oxygen and 5% CO_2_. Cells were grown in ɑ-MEM (Hyclone, USA) which was supplemented with 10% fetal bovine serum (FBS, Hyclone, USA) and 1% antibiotics (100 U/mL penicillin and 100 μg/mL streptomycin). L6 cells were induced differentiation by replacing ɑ-MEM containing 2% FBS for 7 days to form myotubes. During the culturing period, the medium was replaced every 48 h. Cell experiments were performed in differentiated myotubes. 3T3-L1 mouse embryo fibroblasts were also cultured in ɑ-MEM which was supplemented with 10% fetal bovine serum and 1% antibiotics. And the cells were then induced to differentiate according to the method reported previously [20].

### GLUT4 translocation assay

The GLUT4 translocation assay was conducted by a cell-based GLUT4 translocation screening system in L6 cells which stably express pIRAP-mOrange. This screening system has been established according to our previous study [21,22]. To be brief, in cellular GLUTs storage vesicles (GSV), IRAP and GLUT4 displayed a strong co-localization reported by many researchers [23,24]. Thus, detecting the IRAP can indirectly reflect the situation of GLUT4 [22]. pIRAP-mOrange cDNAs were inserted into the pQCXIP plasmid, and retrovirus were prepared by transfecting pQCXIP-IRAP-mOrange. After that, L6 cells at the exponential growth phase were infected with fresh prepared viruses. Finally, the single clone which had the highest increase in red fluorescence intensity following stimulation with insulin (100 nM) was selected.

L6 cells which stably expressing IRAP-mOrange were seeded in 48 well plates and incubated until 100% confluence. Before being treated with samples or other agents, cells were starved in serum-free MEM-α for 2 h. The dosage of SMR treatment was 40 μg/mL to the cells, and insulin (10 nM) was treated as a positive control. The cellular fluorescence photographs were taken with a laser-scanning confocal microscope LSM 510 (Carl Zeiss, Jena, Germany) with 555 nm excitation laser to supervise the IRAP-mOrange translocation. The images were captured every 10 s in first 5 mins and then every 5 mins in later 25 mins.

### Glucose uptake

We used a cell-based fluorescently-labeled deoxyglucose analog kit (2-NBDG kit) to detect glucose uptake in L6 myotubes and 3T3-L1 adipocytes. Differentiated L6 myotubes cells and 3T3-L1 adipocytes were seeded in a 96-well black plate at the density 1 × 10^4^–5 × 10^4^ cells/well, and incubated in 100 μL α-MEM medium overnight until confluence. Subsequently, cells were treated with different doses of SMR or insulin in 100 μL glucose-free α-MEM medium containing 150 μg/mL 2-NBDG (Cayman Chemical, USA). After 24 h incubation, plates were centrifuged for 5 minutes at 400 g at room temperature. The supernatant was aspirated, and 200 μL of cell-based assay buffer was added into each well. This procedure was repeated twice to ensure cells washed up. Then 100 μL of assay buffer was added into each well. Subsequently, the 2-NBDG taken up by cells was detected with ﬂuorescent microplate reader (excitation/emission = 485/535 nm).

### Western blotting analysis in L6 cells

Protein extraction and western blotting analysis were performed according to our previous study [21,22]. Briefly, L6 cells were pre-incubated with different dosage of SMR (10, 20, 40 μg/mL) and insulin (10 nM) for 24 h before extracting proteins for western blot analysis. Then the differentiated cells were harvested and lysed in ice-cold lysis buffer for 30 min, followed by centrifugation at 10,000*g* for 15 min at 4°C. Total protein concentrations were measured by using the BCA kit (Bio-Rad Laboratories, Munich, Germany). Equal amount of protein (70 μg) was subjected to SDS−polyacrylamide gel electrophoresis to be separated and transferred to PVDF membranes (Millipore Corp., Bedford, MA, USA). Then the membranes were blocked with 5% evaporated milk and incubated with primary antibodies GLUT4 and β-Actin (Cell Signaling Technology., Danvers, MA, USA) at 4°C overnight. The membranes were washed out for three times with washing buffer, and subsequently incubated with HRP-conjugated secondary antibodies (Cell Signaling Technology, Beverly, MA, USA) for 1 h at room temperature. Eventually, the immunoreactive signals were measured by enhanced chemiluminescence kits (Amersham-Pharmacia, Piscataway, NJ, USA).

In order to test the time that SMR can take effects on increasing GLUT4 content on L6 cell membrane, we performed western blot analysis to detect GLUT4 level on L6 cell membrane which incubated with single dosage (40 μg/mL) but different time (0 min, 20 min, 30 min and 24 hrs) of SMR. Briefly, to prepare the plasma membrane and post-plasma membrane fraction, L6 myotubes were homogenated, and each homogenate was centrifuged at 1000g for 10 min at 4°C. The precipitate was re-suspended in buffer A containing 1.0 % (v/v) IGEPAL CA-630, placed on ice for 1 h with occasional mixing and was centrifuged at 16,000g for 20 min at 4°C. The supernatant was collected and stored as the plasma membrane (PM) fraction at –80 °C until analyses.

### Animals and treatments

KK-Ay mice are obtained from a cross of black KK females with obese yellow Ay males, which are obese, hyperglycemic, hyperinsulinemic, and insulin resistant [25,26]. In our study, we choose KK-Ay mice as a model of metabolic disorders to evaluate the anti-diabetic effects of SMR. C57BL/6J is the genetic background of KK-Ay mice, so that it is commonly used as normal control group in antidiabetic researches when KK-Ay mice are used as type 2 diabetic model mice [27]. 8-week-old male diabetic KK-Ay mice and their isogenic line 8-week-old normal male C57BL/6J mice were all purchased from Beijing HFK Bioscience Co, Ltd. (Certification number: SCXK 2009-0015). Mice were separately housed in environmentally controlled conditions maintained a 12 h light/12 h dark cycle, temperature 23 ± 3°C with a relative humidity of 55 ± 15%, and allowed free access to food and water. C57BL/6J mice were given a standard diet as normal control while the KK-Ay mice were given a high-fat diet purchased from Medicience Co., Ltd., Yangzhou, China. The composition of the high-fat diet was as follows: protein, 225 g/kg; fat, 200 g/kg; carbohydrate substances, 450 g/kg; cholesterol, 12.5 g/kg; sodium cholate, 5 g/kg; energy, 4500 kcal/kg. After 4 weeks sustaining feeding with high fat diets, the fasted blood glucose (FBG) levels of KK-Ay mice were tested. FBG levels were measured weekly by a blood glucose meter (OneTouch Ultra®, Lifescan Inc., Wayne, USA). Mice with a stable blood glucose level ≥ 11.1 mmol/L were identified as T2DM models and were considered suitable for the following experiments. The successfully established diabetic KK-Ay mice (*n* = 41) were randomly divided into four groups: vehicle group (saline treatment, *n* = 10); SMR-treated groups (50 mg/kg/day, *n* = 10; 100 mg/kg/day, *n* = 11). As we all known, metformin is a commonly used anti-diabetic drug which has been demonstrated can increasing GLUT4 expression and translocation through activating AMPK pathway [28,29]. So we choose metformin as a positive control drug (200 mg/kg/day, *n* = 10). C57BL/6J mice (*n* = 10) were treated with saline as a normal control group in this experiment. SMR was made into a suspension with saline containing 0.5% CMC-Na (sodium carboxymethylcellulose). Metformin was dissolved with the same saline containing 0.5% CMC-Na. Normal control group and vehicle group were intragastric administration with the same solvent. All groups received four consecutive weeks of intragastric administration. The volume of intragastric administration was 0.1 mL/10 g to each mouse. Body weight and food intake were recorded daily. FBG levels were measured every other week. Oral glucose tolerance test (OGTT) (2.0 g/kg glucose) was performed in overnight-fasted mice as described before [21,22]. The area under the curves (AUC) generated from the data collected during the OGTT was calculated. After 4 weeks of treatment, all animals were fasted overnight and sacrificed with all blood samples collected. And we also collected liver, limbs skeletal muscle, pancreas, and subcutaneous WAT stored at −80°C.

### Ethics statement

The research project was approved by the Ethical Committee at South-Central University for Nationalities (SCUN) and all procedures for the use and care of animals for this research were carried out under the approval by the Ethical Committee of Experimental Animal Care at SCUN (approval no. 2016-SCUEC-AEC-0027).

### Biochemical and histological analysis

Each serum sample was obtained by centrifuging the blood samples at 3000 rpm for 15 min. The serum biochemical indexes including total cholesterol (TC), triglycerides (TG), low density lipoprotein cholesterol (LDL-C), and high density lipoprotein cholesterol (HDL-C) were evaluated by an automatic biochemical analyzer (Hitachi 7180+ISE, Tokyo, Japan). Free fatty acid (FFA) was determined by corresponding assay kits (Jiancheng Bioengineering Institute, Nanjing, China). Plasma insulin content was measured by using a rodent insulin enzyme-linked immunosorbent assay (ELISA) kit (Linco Research, St. Charles, MO). TC, TG, and FFA in liver and skeletal muscle were determined with corresponding assay kits (Jiancheng Bioengineering Institute, Nanjing, China) according to the method described previously [21,22]. Partial liver, pancreas, and WAT were fixed with 10% neutral-buffered formalin and embedded in paraffin; 3–4 μm thickness sections were taken with a tissue processor, and stained with HE solution for microscopy. The stained tissues were photographed through an optical microscope photographed (200×).

### Tissue extracts and western blotting

GLUT4 expression level in tissues was detected by using western blotting technique. The skeletal muscle tissue and WAT were chopped into small pieces and homogenized in ice-cold RIPA buffer (50 mM Tris-HCl (pH 7.4), 150 mM NaCl, 1% NP-40, 0.1% SDS) containing 0.1% protease inhibitor (Beyotime, Nanjing, China). The protein solutions were collected from the supernatant after centrifugation at 12,000*g* for 15 min. The concentration of protein contents was determined BCA assay kit (Beyotime, Nanjing, China). Then the following western blotting steps were according to the section described above.

### Statistical analysis

Differences between groups were analyzed with one-way analysis of variance (ANOVA) or one-way repeated measures ANOVA, followed by Tukey’s post hoc test using Graphpad prime 5.0 software. All data were shown as means ± standard error (M ± SEM). A probability (*p*) value of less than 0.05 was considered statistically significant.

## Results

### Effects of SMR on GLUT4 translocation and expression and glucose uptake assay in L6 cells

The effect of SMR on GLUT4 translocation was assessed in L6 cells which stably expressed IRAP-mOrange. As shown in Figure 1(b,c), SMR treatment increased fluorescence intensity on L6 cell membranes with a time-dependent manner. The fluorescence reached the greatest intensity (1.44-fold) at 20 minutes after addition of SMR. These results showed that SMR had a medium effect on the GLUT4 translocation activity in L6 cells. At the same time, the effect of SMR on glucose uptake was detected in L6 cells. SMR treatment increased glucose uptake in a concentration-dependent manner (Figure 1(d)). SMR greatly stimulated glucose uptake at the concentration of 10, 20, and 40 μg/mL. The effect of SMR was further investigated on GLUT4 protein expression by performing western blotting assays in L6 myotubes. As shown in Figure 1(e,f), after incubation with SMR, the GLUT4 expression was increased significantly in L6 cells compared with normal control.

From Figure 1(h,i), we can conclude that SMR displayed the good effects on increasing GLUT4 content on membrane in L6 cells with different incubation time. Even 24 hrs later with SMR incubation, the GLUT4 content on L6 cell membrane is still high.

### SMR reduces body weights of HFD-induced KK-Ay mice

**Figure 2.**
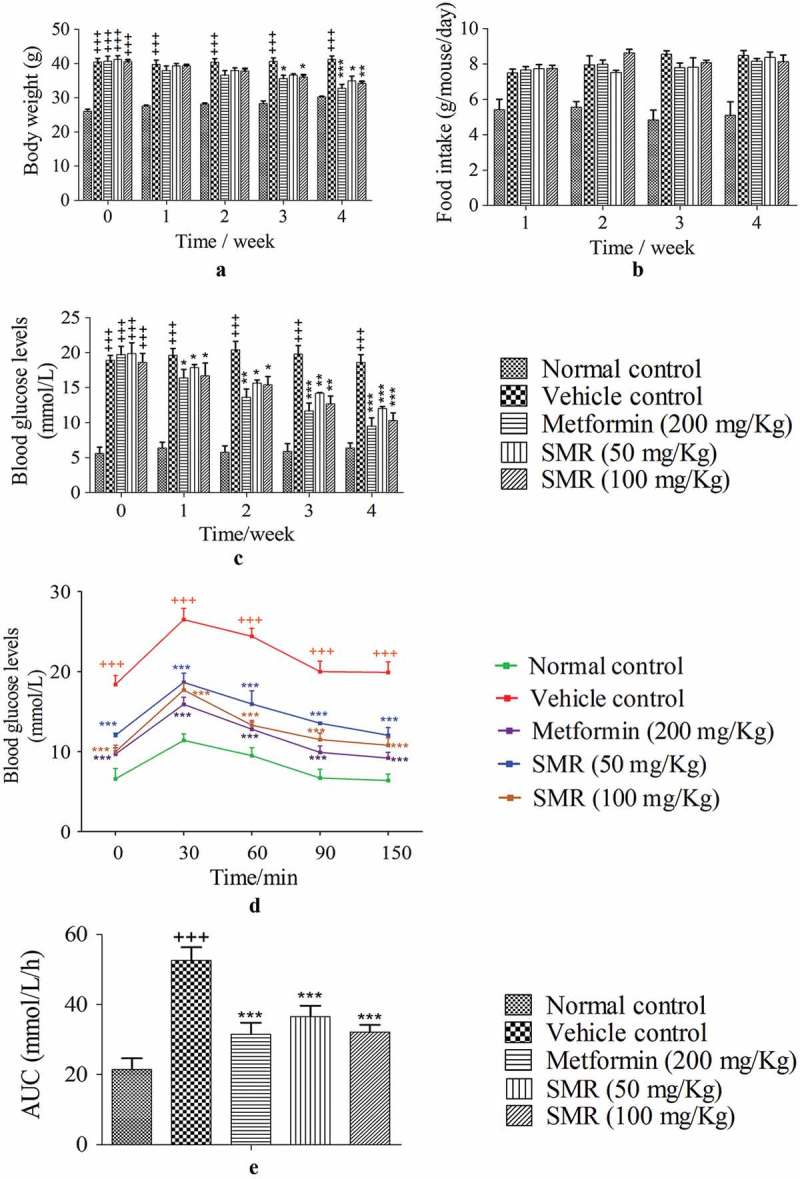
The effects of SMR on body weight, food intake, serum glucose level and OGTT. (a) The effects of SMR on body weight reduction. (b) Food intake of all groups during four consecutive weeks. (c) The effects of SMR on decreasing FBG levels. (d) The effects of SMR on OGTT after 4-week treatment. (e) AUC of the OGTT after 4-week treatment of SMR (*n* ≥ 10 mice in each group, ^+++^ *p* < 0.001 compared with normal control, *p < 0.05, **p < 0.01, ***p < 0.001 compared with vehicle control).

The body weights of KK-Ay mice were visibly higher than those of the normal C57BL/6J mice in the initial before our treatment. As the experiment went on, the effects of SMR on reducing body weight had started to appear. As the results shown in Figure 2(a), there was no significant difference in the initial body weight between treated groups and vehicle group. But the body weights of mice in SMR-treated groups and metformin-treated group were gradually reduced by consecutive weeks. After 4 weeks treatment, body weights of each treated group exhibited a significant difference compared to those of the vehicle group. Although the daily energy intake of the KK-Ay mice groups were significantly higher than that of the normal control group, two SMR treated groups and metformin treated group did not affect the daily energy intake of KK-Ay mice during four weeks (Figure 2(b)).

### SMR attenuates hyperglycemia in HFD-induced KK-Ay mice

As shown in Figure 2(c), at the start of the experiment, FBG levels were significantly higher in KK-Ay diabetic model mice (FBG ≥ 18.5 mmol L^−1^, *p* < 0.001) compared to the normal mice (5.2 ± 0.7 mmol L^−1^). However, after metformin-, low-, and high-dose SMR treatment, FBG levels in KK-Ay mice were decreased compared to that in vehicle control mice. After 4 weeks of treatment, the FBG levels were decreased to 9.8 ± 1.3 mmol L^−1^, 12.1 ± 1.7 mmol L^−1^, and 10.7 ± 1.5 mmol L^−1^, respectively, in metformin-, low-, and high-dose SMR treated groups. While the FBG level in the vehicle control mice kept still extremely high to 18.1 ± 2.6 mmol L^−1^. This result demonstrated the beneficial effect of SMR in reducing blood glucose in KK-Ay mice. At the end of the experiments, an OGTT test was performed (Figure 2(d)). Compared with the normal C57BL/6J mice, the vehicle group mice showed a sharp increase in plasma glucose levels after oral administration of 2.0 g/kg glucose, and the elevated blood glucose levels were much higher than starting levels even at 150 minutes, suggesting a decline in the glucose tolerance of KK-Ay diabetic mice. However, SMR-treated groups and metformin-treated group blocked an increase of blood glucose levels in KK-Ay mice at any time point after oral glucose administration, and the elevated blood glucose levels were reduced quickly in those mice. The OGTT results indicated a significant improvement in impaired glucose tolerance.

### Effects of SMR on serum and tissue lipid levels

**Figure 3.**
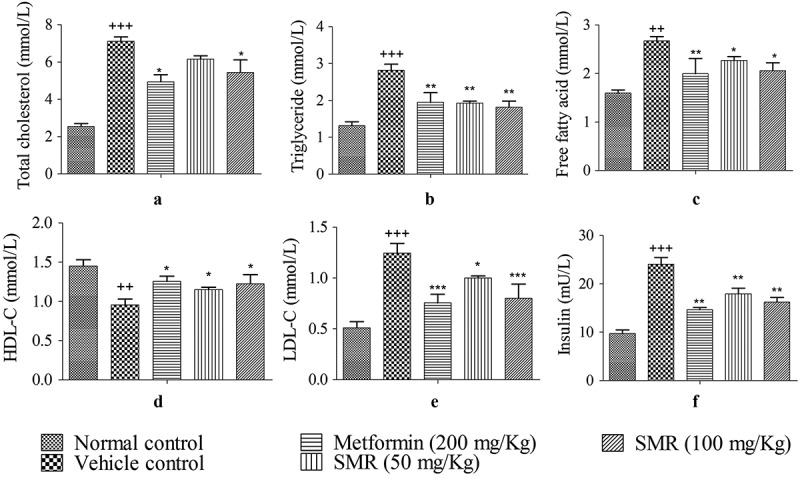
Effects of SMR on serum lipid levels and insulin level. (a–e) Effects of SMR on TC, TG, FFA, HDL-C and LDL-C levels in the serum. (f) Effect of SMR on serum insulin level (*n* ≥ 10 mice in each group, ^+++^ *p* < 0.001, ^++^ *p* < 0.01 compared with normal control, *p < 0.05, **p < 0.01, ***p < 0.001 compared with vehicle control).

**Figure 4.**
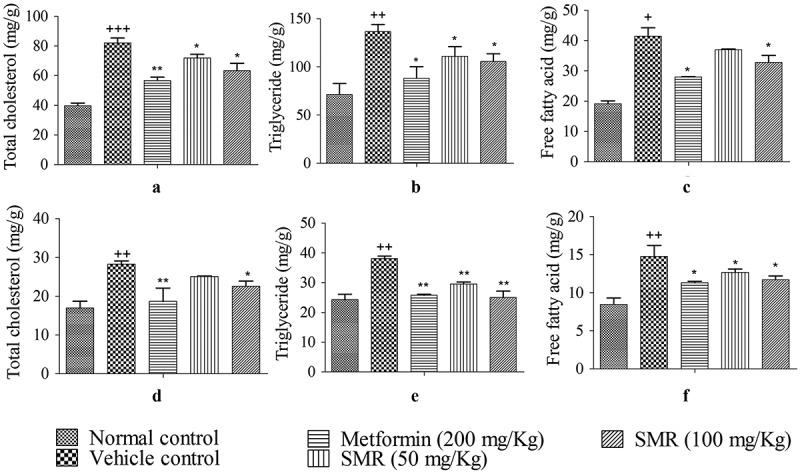
Effects of SMR on liver and skeletal muscle lipid levels. (a–c) Effects of SMR on TC, TG and FFA levels in liver. (d–f) Effects of SMR on TC, TG and FFA levels in skeletal muscle (*n* ≥ 10 mice in each group, ^+++^ *p* < 0.001, ^++^ *p* < 0.01 compared with normal control, *p < 0.05, **p < 0.01 compared with vehicle control).

Serum lipid indexes were abnormal in KK-Ay mice rather than C57BL/6J mice shown in Figure 3(a–e). When treated with SMR and metformin for 4 weeks, the metabolic symptoms of KK-Ay mice tended to be improved. The concentrations of TG, TC, FFA and LDL-C in treated groups were significantly lower than those in the vehicle group, whereas the concentration of HDL-C was markedly higher in the treated groups than in the vehicle group. IR is one of the most common characteristics in type 2 diabetes. The serum insulin levels were detected in each group mice. The results showed that serum insulin levels were significantly increased in KK-Ay mice than those in normal mice in Figure 3(f). After 4-week treatment with SMR, the insulin levels were reduced compared with those in the vehicle group. Meanwhile, the same effects were found in metformin treated group. The contents of TG, TC and FFA in liver and skeletal muscle were much higher in KK-Ay mice groups than that in C57BL/6J mice group (Figure 4). After 4 weeks treatment with SMR, the contents of TC, TG and FFA tended to be declined in liver and muscle compared with the un-treated KK-Ay group, and the changes were always significant. The positive control group treated with metformin had a most remarkable effect on changing the abnormal lipid metabolism in KK-Ay mice.

### Histological examination in pancreas, liver and WAT

**Figure 5.**
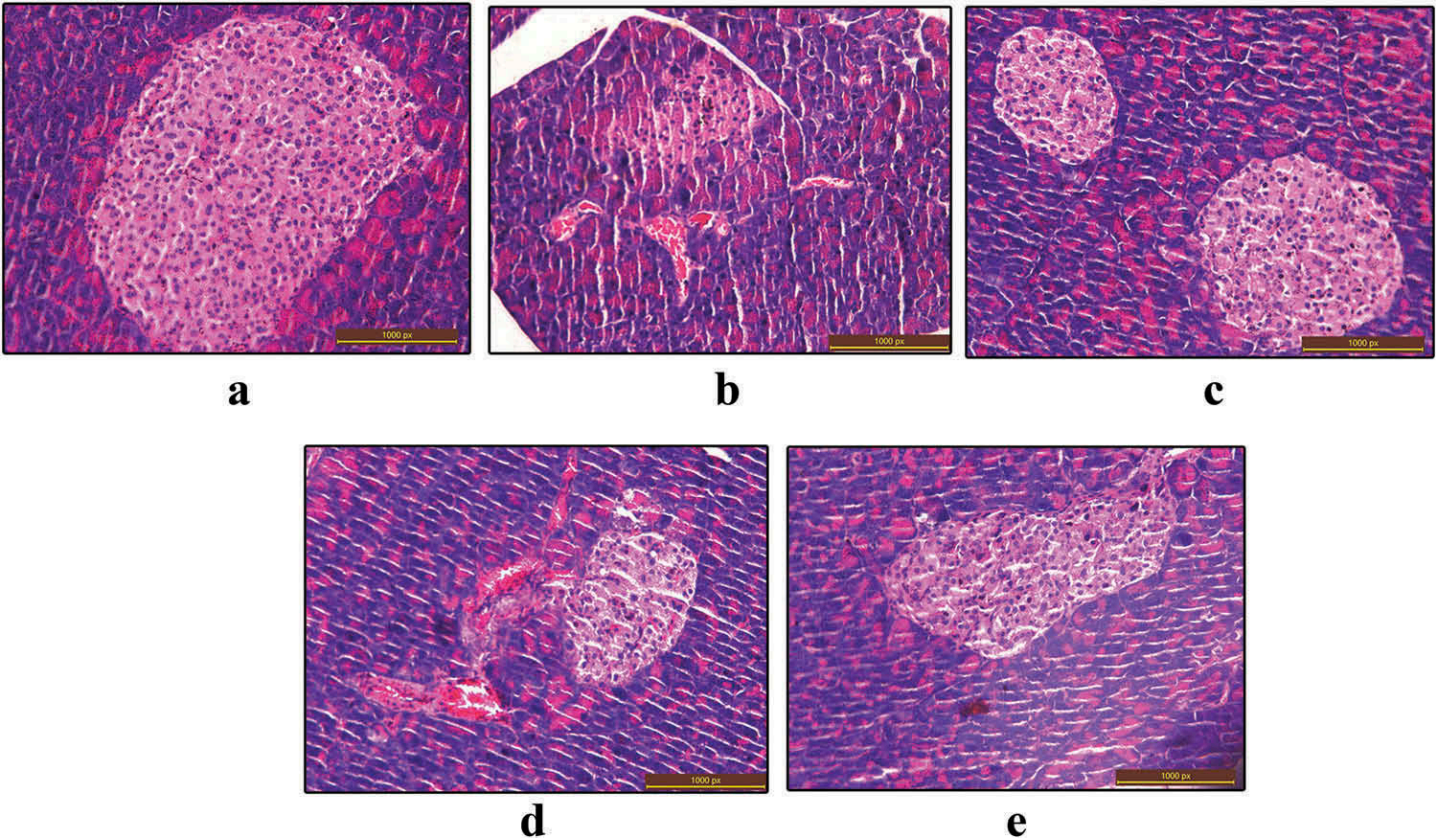
Effects of SMR on pancreas protection in KK-Ay mice. Optic microscopy: HE (200×). (a) Normal control. (b) KK-Ay mice treated with vehicle. (c) KK-Ay mice treated with metformin (200 mg/kg). (d) KK-Ay mice treated with SMR (50 mg/kg). (e) KK-Ay mice treated with SMR (100 mg/kg).

The sections of KK-Ay mouse pancreas were performed as previously described in the methods section. In the normal control group, pancreatic section showed normal islets, normal pancreatic structure and size (Figure 5(a)). In the vehicle group, pancreatic section showed a disorganization of the structure and smaller size of islet compared with the normal control group. However, in SMR- and metformin-treated groups, the KK-Ay mouse pancreas was less damaged compared with the vehicle group (Figure 5(b–e)). And the protection on pancreas was more significant in 100 mg/kg SMR-treated group than the effects of 50 mg/kg SMR-treated group.

**Figure 6.**
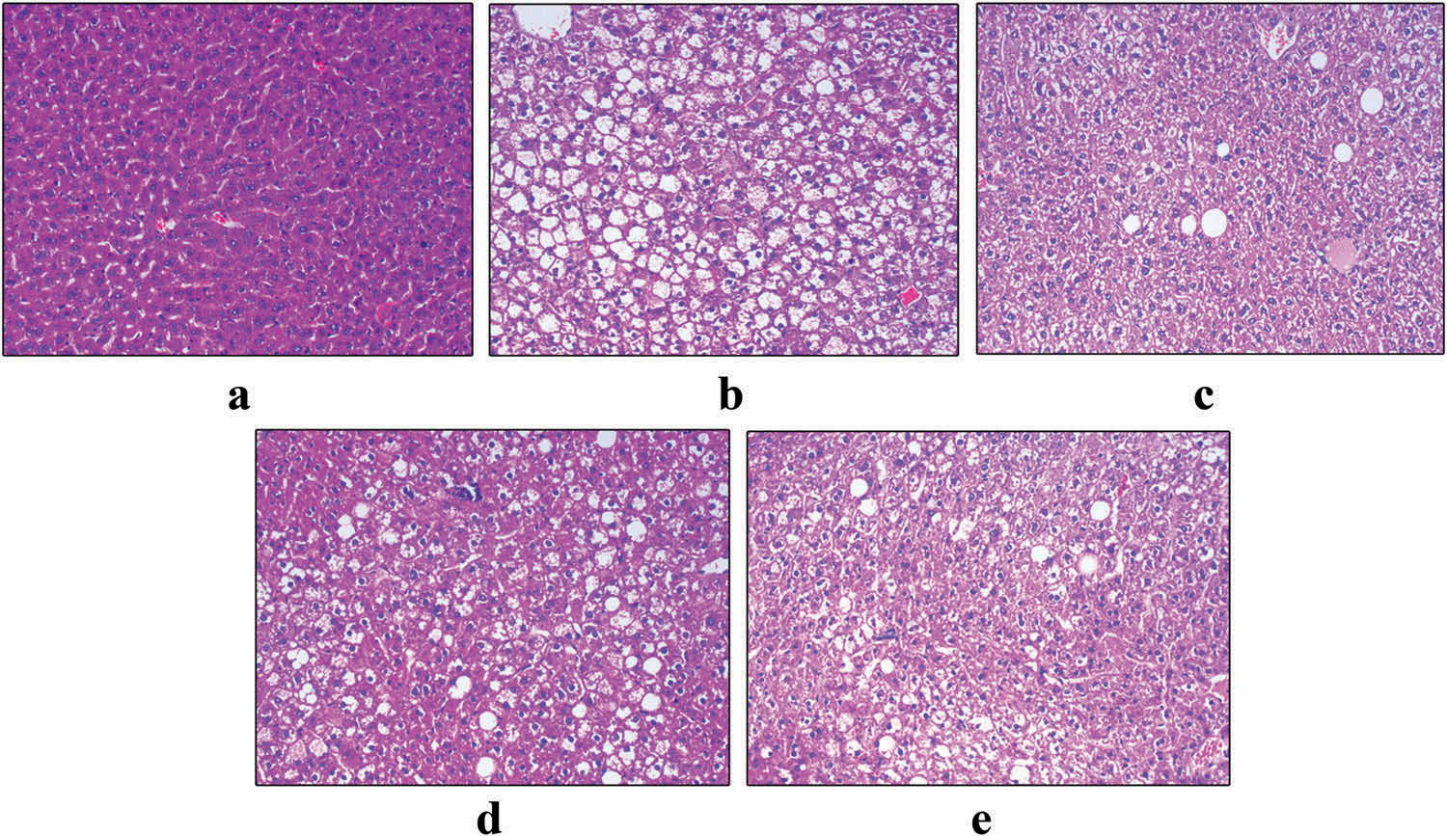
Effects of SMR on morphological features of mice livers. Optic microscopy: HE (200×). (a) Normal control. (b) KK-Ay mice treated with vehicle. (c) KK-Ay mice treated with metformin (200 mg/kg). (d) KK-Ay mice treated with SMR (50 mg/kg). (e) KK-Ay mice treated with SMR (100 mg/kg).

Hepatic steatosis and empty lipid vacuoles were observed in the KK-Ay model mice, whereas C57BL/6J mice liver section showed normal cell morphology (Figure 6). By contrast, few lipid droplets and the reduction of hepatic intracellular lipid load were observed in KK-Ay mice treated with SMR and metformin. The results illustrated that SMR alleviated hepatic steatosis in different degrees.

**Figure 7.**
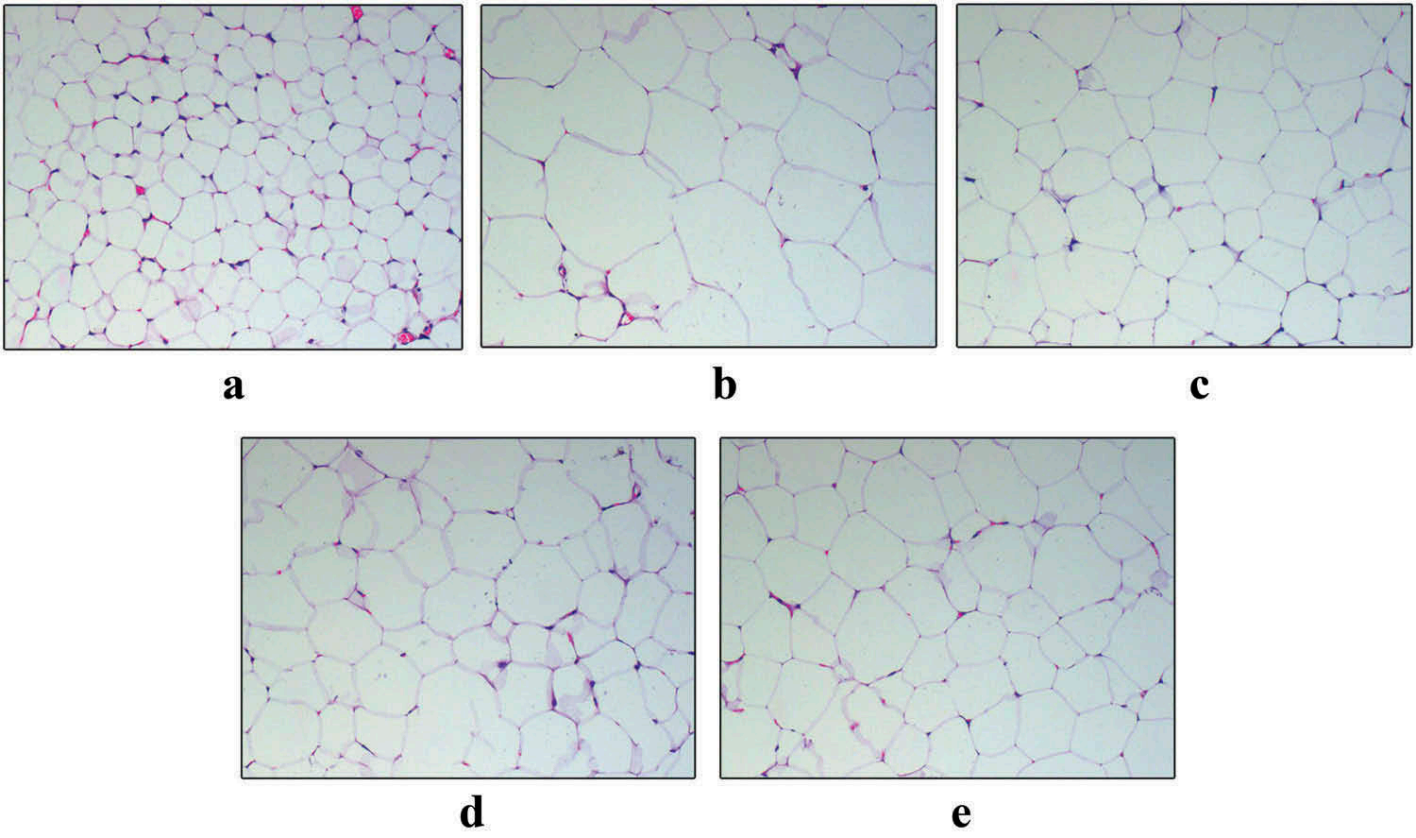
Chronic oral treatment with SMR decreased WAT size in KK-Ay mice. Optic microscopy: HE (200×). (a) Normal control. (b) KK-Ay mice treated with vehicle. (c) KK-Ay mice treated with metformin (200 mg/kg). (d) KK-Ay mice treated with SMR (50 mg/kg). (e) KK-Ay mice treated with SMR (100 mg/kg).

The effect on reducing body weight in KK-Ay mice caused by SMR interested us, so we also performed an HE staining to examine the morphology of WAT. The bigger adipose cell size was observed in KK-Ay mice compared with those in normal control group (Figure 7(a)). However, after treatment with SMR (50 mg/kg), SMR (100 mg/kg), and metformin, the adipocyte size in three treated groups were significantly smaller than those in the vehicle group (Figure 7(b–e)).

### GLUT4 expression in skeletal muscle and WAT

**Figure 8.**
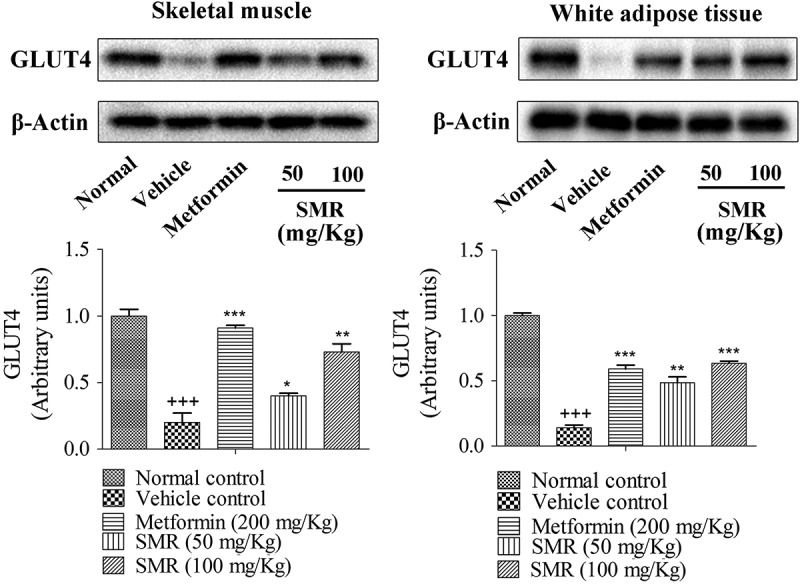
Effects of SMR on GLUT4 expression in skeletal muscle and WAT (data shown as relative band intensity compared with normal mice, ^+++^ *p* < 0.01 compared with normal control, *p < 0.05, **p < 0.01, ***p < 0.001 compared with vehicle control).

In the further mechanism study, we conducted western blot analysis in skeletal muscle and WAT of KK-Ay mice. The results showed that the levels of GLUT4 of skeletal muscle and WAT collected from KK-Ay mice were lower than those from normal C57BL/6J mice (Figure 8). However, the reduction was significantly improved after 4 weeks treatment with SMR or metformin. In skeletal muscle, the expressions of GLUT4 significantly increased to 1.1 fold and 2.4 fold, respectively, in 50 and 100 mg/kg SMR-treated groups compared with those in vehicle groups. The same activating GLUT4 expression effects were observed in WAT caused by SMR. After 4 weeks treatment with SMR, GLUT4 expressions were enhanced in WAT.

## Discussion

In search of new drugs to fight against IR and type 2 diabetes (T2DM), compounds from natural foods have drawn more and more attention [30,31]. Many chemical compounds derived from natural foods have been reported to exert strong biological activity against T2DM [30,31]. Among of them, plant sterols and stanols have drawn particular attention due to their wide distribution in food plants and potent ability to reduce blood fat content [12]. In the present study, we mainly investigated the effects of SMR, which is one kind of plant sterol involved in T2DM, and its possible mechanisms. The results showed that SMR significantly increased the GLUT4 translocation and glucose uptake in L6 cells. Further *in vivo* study demonstrated that the hyperglycemic phenotype of diabetic KK-Ay mice were significantly alleviated after 4 weeks of SMR treatment, at which point significantly reduced fasting glucose was observed, indicating a notable hypoglycemic effect against T2DM. Levels of serum insulin and oral glucose tolerance were also significantly lower in the treated groups than in the untreated KK-Ay mice, showing IR had been relieved by SMR to some extent

IR, defined as the reduced response of cells or tissues to physiological levels of insulin and enhanced blood insulin in patients, is a remarkable feature of T2DM [32]. For many years, IR has been seen as the core defect responsible for the development of T2DM [33]. An abundance of evidence has demonstrated that chronic tissue inflammation caused by obesity is the most common etiology of obesity-induced IR [34]. Obesity, hyperinsulinemia, and hyperlipidemia are often coupled with T2DM, indicating that hyperglycemia and hyperlipidemia will never develop if IR is overcome [35]. In the present study, SMR significantly improved IR and lipid metabolism in KK-Ay mice. After 4 weeks of treatment with SMR, the FBG level and body weight had been reduced, the serum insulin concentration had been dramatically reduced, and the OGTT showed that the sensitivity of insulin had been visibly improved by SMR. The biochemical indexes of blood lipid metabolism (such as HDL-C, LDL-C, FFA, TG, and TC) had also been improved at different degrees.

Muscle, adipose, and liver are the three types of tissue most profoundly affected by peripheral IR [36]. IR leads to severely dysregulated glucose and lipid homeostasis, resulting in a worsening of insulin insensitivity in these organs, eventually leading to lipid accumulation [37]. In our study, the lipid accumulation in the liver and the bigger adipocyte size were observed in KK-Ay model mice. However, after 4 weeks of treatment with SMR, the treated mice presented reduced lipid vacuoles in the liver and smaller adipocytes in WAT. At the same time, the biochemical indexes of TC, TG, and FFA in the liver and skeletal muscle were ameliorated at different degrees, corresponding closely to the morphological improvements by SMR. During the 4-week treatment with SMR, damage to pancreas was alleviated in the KK-Ay mice to some degree. As is well known, IR plays a vital role in insulin secretory dysfunction and apoptosis of functional β-cells in the progress of T2DM [38]. Our results showed that SMR prevented a decrease in pancreas islet size and normalized the forms of islets. These findings collectively showed that SMR could target the fundamental cause of IR and thereby contributed to the overall improvement of metabolic features in T2DM animals.

The insulin-responsive facilitative glucose transporter GLUT4 is one of the 13 known glucose transporter proteins (GLUT1–GLUT12 and HMIT), and it is present mainly in adipose, skeletal muscle, and cardiac muscle cells [39,40]. GLUT4 translocation and expression levels are correlated with whole-body insulin-mediated glucose homeostasis [41,42]. More and more attention has been drawn to the link between IR and GLUT4, and some interesting conclusions have been made [43]. Berger et al., Cushman et al., and Sivitz et al. have reported that insulin-resistant glucose transport in adipocytes from obese and diabetic subjects is correlated with reduced GLUT4 mRNA and protein expression, confirming a role of GLUT4 for insulin-dependent glucose homeostasis [43–45]. Treadway et al. also reported that transgenic manipulation of GLUT4 in mice has a profound effect on both glucose and lipid homeostasis [45]. In this way, a moderate increase in the expression of GLUT4 is a good target for the treatment of IR in patients. In our present study, we established a L6-based GLUT4 translocation system to discover natural agents that may promote GLUT4 translocation to the cell membranes [46,47]. In an *in vitro* study, SMR exhibited strong stimulation of GLUT4 transportation onto plasma membranes. The expression of GLUT4 in L6 cells was also significantly elevated after adding SMR. These *in vitro* results provide preliminary evidence of the role of SMR in enhancing GLUT4 expression. In severe IR, GLUT4 protein expression can be reduced similarly both in adipose tissue and skeletal muscle. Fat-specific and muscle-specific knockout of GLUT4 affects whole-body glucose homeostasis and leads to IR [48,49]. Further verification testing was conducted in the skeletal muscle and WAT of KK-Ay mice. As expected, the *in vivo* results showed that the GLUT4 expression levels in the skeletal muscle and WAT were dramatically lower than in the normal C57BL/6J mice. However, after 4 weeks treatment of SMR, GLUT4 expression levels in these tissues were improved significantly. Collectively, all of the present findings concerning GLUT4 expression and translocation both *in vitro* and *in vivo*, demonstrated that SMR may contribute to enhancing the basal expression of GLUT4 in cells and partial correction of the defect in insulin-mediated GLUT4 translocation, thereby mitigating IR in KK-Ay mice.

In conclusion, SMR, a kind of plant sterols derived from soil bean oil, effectively ameliorated the hyperglycemia and hyperlipidemia in KK-Ay mice. Moreover, the possible antidiabetic mechanism of SMR was elucidated that SMR improved IR by enhancing GLUT4 expression and translocation *in vitro* and *in vivo*. Based on these findings, SMR has the potential to become an effective agent in the therapy of T2DM.

## Supplementary Material

Revised_SI_ZFNR_1364117.doc
