# Supplementary material for: Anti-diabetic activity of stigmasterol from soybean oil by targeting the GLUT4 glucose transporter
Source: Food Nutr Res. 2017 Aug 23;61(1):1364117. doi: 10.1080/16546628.2017.1364117 (PMC5614214; doi:10.1080/16546628.2017.1364117)
Supplement: Revised_SI_ZFNR_1364117.doc [file ZFNR_A_1364117_SM2975.doc]

**Supporting Information**

**Anti-diabetic activity of stigmasterol from soybean oil by targeting GLUT4 glucose transporter**

Jialin Wang^a,1^, Mi Huang^a,1^, Jie Yang^a^, Xinhua Ma^a^, Sijian Zheng^a^, Shihao Deng^a^, Yun Huang^a^, Xinzhou Yang^a,c*^ Ping Zhao ^b,*^

^a^ School of Pharmaceutical Sciences, South-Central University for Nationalities, Wuhan 430074, China

^b^ College of Life Sciences, South-Central University for Nationalities, Wuhan 430074, China

^c^ National Demonstration Center for Experimental Ethnopharmacology Education, South-Central University for Nationalities, Wuhan 430074, China

Corresponding authors at: School of Pharmaceutical Sciences, South-Central University for Nationalities, 182 Min-Zu Road, Wuhan 430074, P.R. China. Tel.: +86 27 67841196; Fax: +86 27 67841196

E-mail addresses: xzyang@mail.scuec.edu.cn (X.Z. Yang); p.zhao@scuec.edu.cn (P. Zhao).

^1^Both authors contributed equally to this paper.

**Abstract**

The present study investigated the anti-diabetic activity and potential mechanism of stigmasterol (SMR) which is a kind of phytosterols derived from the edible soybean oil in vitro and in vivo. SMR displayed a mild GLUT4 translocation activity by 1.44-fold in L6 cells. L6 cells were treated with different concentration of SMR, showing significant effects on the enhancing glucose uptake. SMR administrated orally to the KK-Ay mice significantly alleviated their insulin resistance and oral glucose tolerance with reducing fasting blood-glucose levels and blood lipid indexes such as triglyceride and cholesterol. Moreover, the GLUT4 expression in L6 cells, skeletal muscle and white adipose tissue (WAT) had been also enhanced. In this paper we conclude that, stigmasterol seems to have potential beneficial effects on the treatment of type 2 diabetes mellitus (T2DM) with the probable mechanism of targeting GLUT4 glucose transporter included increasing GLUT4 translocation and expression.

**Keywords**: Anti-diabetic agents; Stigmasterol; L6 cells; KK-Ay mice; Insulin resistance; GLUT4

**Supporting Information Contents**

**S1. Screening methodology validation**

**S2. ^1^H-NMR spectrum of stigmasterol**

**S3. ^13^C-NMR spectrum of stigmasterol**

**S4. ESIMS spectrum of stigmasterol**

**S1. Screening Methodology Validation**

There have several papers reported that IRAP-mOrange and GLUT4-eGFP could be applied to detect the GLUT4 translocation in L6 (Wang et al., 2009; Zhou et al., 2016; Huang et al., 2016) and 3T3-L1 cells (Bai et al., 2007; Jiang et al. 2008). In order to validate the feasibility of our IRAP translocation assay for discovering potential hypoglycemic agents, we have observed the effects when the GLUT4-eGFP or IRAP-marked L6 cells treated with insulin and berberine which are definitely pharmacodynamic GLUT4 agonists. L6 cells which stably express IRAP-mOrange and GLUT4-eGFP were cultured in MEM-ɑ supplemented with 10% fetal bovine serum and 1% antibiotics (100 U/mL penicillin and 100 μg/mL streptomycin) at 37 ^o^C in 5% CO2. L6 cells was seeded in 48 well plates, and incubated until 100% confluence and then starved in serum-free MEM-ɑ for 2 h. Afterwards, L6 cells were treated with insulin (10 nM) and berberine (5 μM). The cells were taken photos with a laser-scanning confocal microscope LSM 510 (Carl Zeiss, Jena, Germany) to supervise the IRAP-mOrange and GLUT4-eGFP translocation. And the images were captured with 555 nm excitation laser every 10 seconds in first 5 minutes and then every 5 minutes in later 30 minutes.

During the experiment, as time went on, we could observe the green and red fluorescence enhanced significantly after treating with insulin and berberine in L6 cells (Fig. 1). The results showed that GLUT4 and IRAP simultaneously translocated onto the plasma membrane in 30 min when adding the GLUT4 agonist. GLUT4 has mainly been recruited to the PM throughout to the GLUTs storage vesicles (GSV). Three main proteins stored in GSV are GLUT4, IRAP, and Sortilin (Shi et al., 2005). It was reported that IRAP and GLUT4 displayed a strong colocalization (Kumar et al., 2010; Rubin et al., 2009). Thus, detecting the IRAP can indirectly reflect the situation of GLUT4. So our results could be explained that detecting the IRAP-mOrange fluorescence could indirectly reflect the GLUT4 translocation. As the red fluorescence is more conspicuous than green fluorescence for observation, so we choose the IRAP-mOrange fluorescence assay for reflecting GLUT4 translocation.


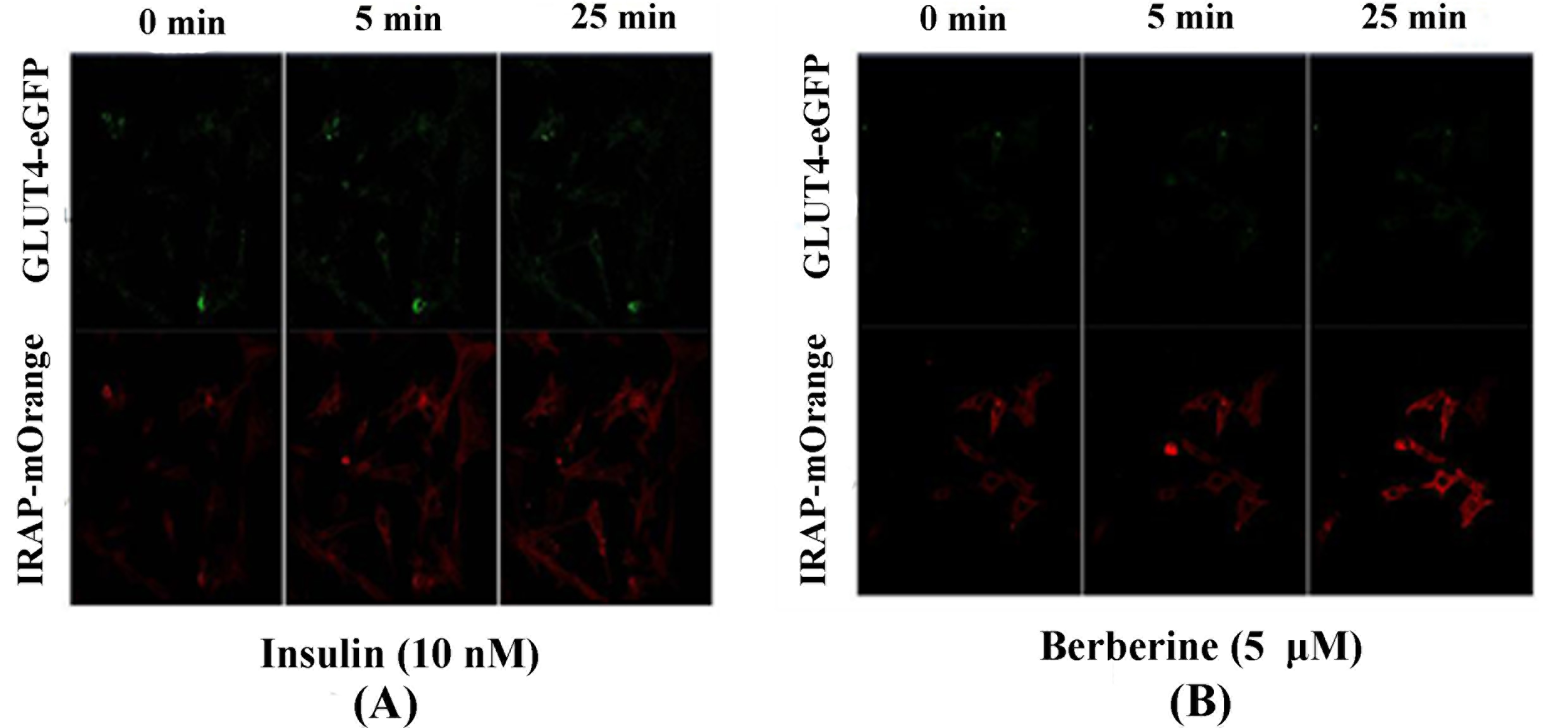


**Figure S1** L6 cells were infected with IRAP-mOrange and GLUT4-eGFP in order to detect externalized GLUT4 translocation by confocal microscopy. (A) Confocal images in L6 cells incubated in the absence (0 min) or presence of insulin for 5min, 30 minutes. (B) Confocal images in L6 cells incubated in the absence (0 min) or presence of berberine for 5 min, 30 minutes.

**References**

Bai, L.; Wang, Y.; Fan, J.; Chen, Y.; Ji, W.; Qu, A.; Xu, P.; James, D. E.; Xu, T. Dissecting multiple steps of GLUT4 trafficking and identifying the sites of insulin action. *Cell. Metab*. 2007, 5, 47-57.

Huang, M.; Zhao, P.; Xiong, M. R.; Zhou, Q.; Zheng, S. J.; Ma , X. H.; Xu, C.; Yang, J.; Yang, X. Z.; Zhang, T. C. Antidiabetic activity of perylenequinonoid-rich extract from Shiraia bambusicola in KK-Ay Mice with Spontaneous Type 2 Diabetes Mellitus. *J. Ethnopharmac.* 2016, 191, 71-81.

Jiang, L.; Fan, J.; Bai, L.; Wang, Y.; Chen, Y.; Yang, L.; Chen, L.; Xu, T. Direct quantification of fusion rate reveals a distal role for AS160 in insulin-stimulated fusion of GLUT4 storage vesicles. *J. Biol. Chem.* 2008, 283, 8508-8516.

Kumar, A.; Lawrence, Jr.; Jung, D. Y.; Ko, H. J.; Keller, S. R.; Kim, J. K.; Magnuson, M. A.; Harris, T. E. Fat cellspecific ablation of rictor in mice impairs insulin-regulated fat cell and whole-body glucose and lipidmetabolism. *Diabetes.* 2010, 59, 1397–1406. doi: 10.2337/db09-1061.

Rubin, B. R.; Bogan, J. S. Intracellular retention and insulinstimulated mobilization of GLUT4 glucose transporters. *Vitam. Horm.* 2009, 80, 155–192. doi: 10.1016/S0083-6729(08)00607-9.

Shi, J.; Kandror, K. V. Sortilin is essential and sufficient for the formation of glut4 storage vesicles in 3T3-L1 adipocytes. *Dev. Cell.* 2005, 9, 99–108.

Wang, X.; Qu, F.; Chen, Z.; Liang, T.; Qu, A. Labeling and imaging of GLUT4 in live L6 cells with quantum dots. Biochem. *Cell. Biol.* 2009, 87, 687-694.

Zhou, Q.; Yang, X. Z.; Xiong, M. R.; Xu, X. L.; Zhen, L.; Chen, W. W.; Wang, Y.; Shen, J. H.; Zhao, P.; Liu, Q. H. Chloroquine Increases Glucose Uptake via Enhancing GLUT4 Translocation and Fusion with the Plasma Membrane in L6 Cells. *Cell Physiol. Biochem.* 2016, 38, 2030-2040.


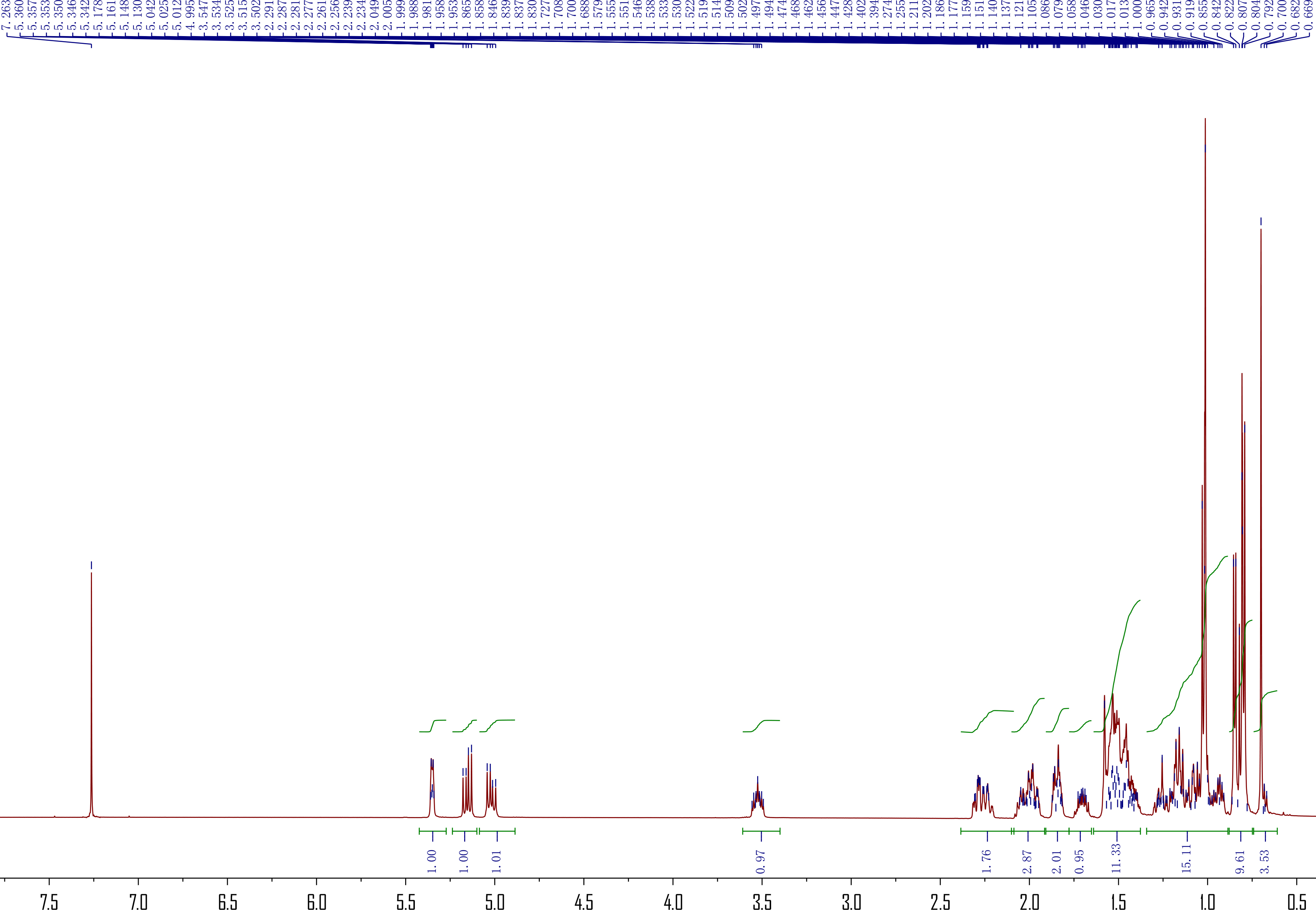


**S2. ^1^H-NMR spectrum of stigmasterol**

**
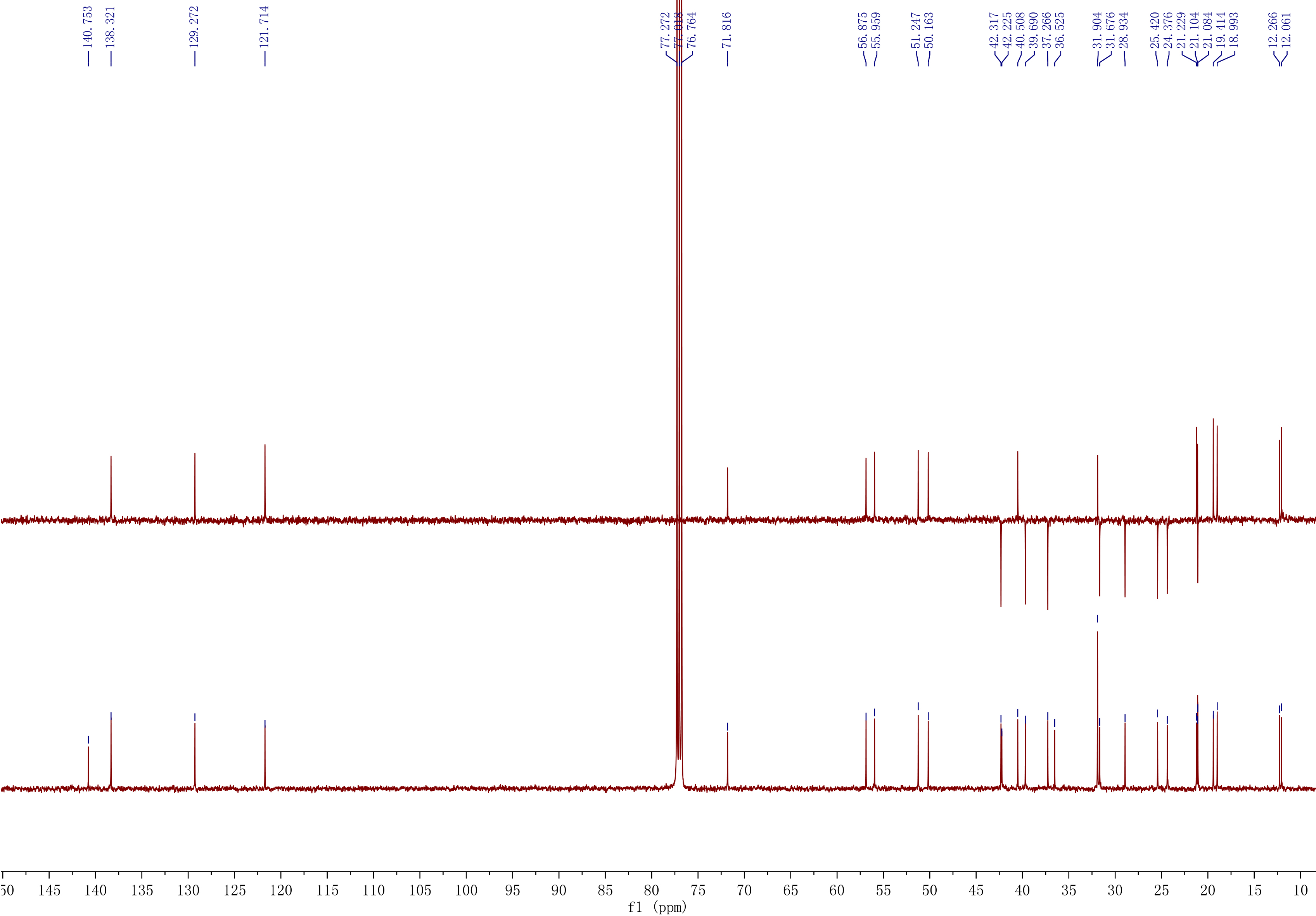
**

**S3. ^13^C-NMR spectrum of stigmasterol**

**
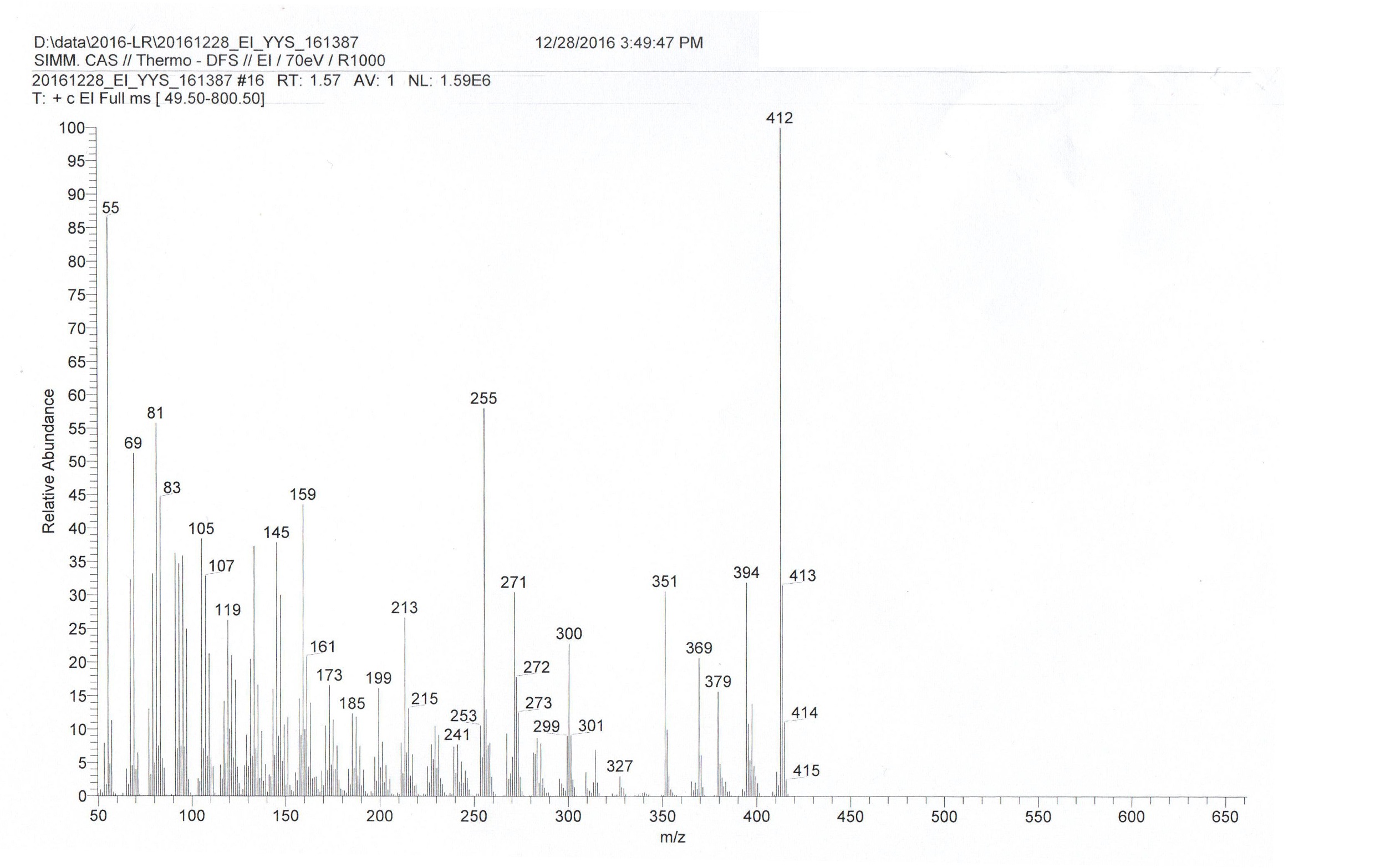
**

**S4. EIMS spectrum of stigmasterol**
